# Adaptation and Evaluation of the Nutrition Environment Measures Survey in Stores to Assess Mediterranean Food Environments (NEMS-S-MED)

**DOI:** 10.3390/ijerph17197031

**Published:** 2020-09-25

**Authors:** Alba Martínez-García, Julia Díez, Carlos Fernández-Escobar, Eva María Trescastro-López, Pamela Pereyra-Zamora, Carles Ariza, Usama Bilal, Manuel Franco

**Affiliations:** 1 Department of Community Nursing, Preventive Medicine and Public Health and History of Science, Universidad de Alicante, 03690 Alicante, Spain; alba.martinez@ua.es (A.M.-G.); eva.trescastro@ua.es (E.M.T.-L.); pamela.pereyra@ua.es (P.P.-Z.); 2 Public Health and Epidemiology Research Group, School of Medicine and Health Sciences, Universidad de Alcalá, Alcalá de Henares, 28801 Madrid, Spain; c.fernandez@isciii.es (C.F.-E.); ub45@drexel.edu (U.B.); manuel.franco@uah.es (M.F.); 3 National School of Public Health, Carlos III Health Institute, 28029 Madrid, Spain; 4 Agència de Salut Pública de Barcelona (Public Health Agency Barcelona), 08023 Barcelona, Spain; cariza@aspb.cat; 5 Ciber de Epidemiología y Salud Pública (CIBERESP), 28029 Madrid, Spain; 6 Institut d’Investigació Biomédica Sant Pau (IIB Sant Pau), 08041 Barcelona, Spain; 7 Urban Health Collaborative and Department of Epidemiology and Biostatistics, Drexel Dornsife School of Public Health, Philadelphia, PA 19104, USA; 8 Department of Epidemiology, Johns Hopkins Bloomberg School of Public Health, Baltimore, MD 21205, USA

**Keywords:** food environment, food retail, measurement, Nutrition Environment Measures Surveys

## Abstract

The Nutrition Environment Measures Surveys are valid and reliable measures of community and consumer food environments. This article describes the adaptation and evaluation of the Nutrition Environment Measures Survey in Stores (NEMS-S) for Mediterranean urban contexts (NEMS-S-MED). Trained raters used the adapted NEMS-S-MED tool to observe and rate food outlets in 21 census tracts and 43 food stores across the city of Madrid, Spain. We evaluated inter-rater and intra-rater reliabilities, construct validity, and the tool’s ability to discriminate between store types and between stores by area-level Socio-Economic Status (SES). Overall, the mean NEMS-S-MED score was 20.7 (SD = 9.8), which ranged from 7 to 43. Most food items displayed substantial or almost perfect inter-rater and intra-rater agreements; the percentage agreement across availability items was almost perfect and kappa statistics were also very high (median κ = 1.00 for inter-rater; κ = 0.92 for intra-rater). Furthermore, the NEMS-S-MED tool was able to discriminate between store types and census tracts of different SES. The adapted NEMS-S-MED instrument is a reliable and valid audit tool to assess the consumer food environment in Mediterranean urban contexts. Well-constructed measurement tools, such as the NEMS-S-MED, may facilitate the development of effective policy interventions to increase healthy food access and affordability.

## 1. Introduction

Unhealthy diets are the leading cause of mortality worldwide [1]. One of the major determinants for unhealthy diets is the food environment [2]. As such, food environments are receiving increasing attention from public health experts and decision-makers as potentially modifiable environmental determinants of diet [3,4].

The retail food environment, as described by the conceptual model of Glanz et al., encompasses the community environment (e.g., type and distribution of food stores) and the consumer environment (e.g., product availability, price, and promotion within stores) [5]. Existing systematic reviews have compiled the evidence on food environment research and have reported that most research has focused on the community food environment [6,7]. Although fewer studies have explored the consumer food environment [8], both healthy food availability and affordability are key influences on dietary behaviors [9]. Moreover, there is evidence for socioeconomic inequalities in food access [9,10]. Yet, this trend is less evident outside the US.

To quantify and assess the consumer food environment, efficient, reliable, and valid measures are needed. Whereas a large number of tools have been developed, few of them have undergone reliability or validity testing [8,11]. To date, the Nutrition Environment Measures Surveys instruments and the USDA Thrifty Food Plan—both developed for the US context—are the most widely used instruments used in health research [11]. The ‘Nutrition Environment Measures Survey in Stores’ (NEMS-S), originally developed by Glanz et al. in 2007, is an observational checklist that assesses the availability and cost of healthier versus less-healthy options based on a mainstream North American diet [12,13]. This audit tool has been shown to have high inter-rater reliability, intra-rater reliability, and face and criterion validity [13]. This tool provides a composite score that can help decision-makers to identify priority areas and inform interventions [14].

Yet, assessing the retail food environment is context-dependent [15]. As such, researchers have adapted the NEMS-S tool for use in several studies conducted in Australia [16], Canada [17], China [18], Brazil [19], or Paraguay [20]. Mediterranean retail food environments present specific characteristics, including trust in small retailers, as opposed to the large reliance of retail chains of supermarkets in the U.S and other Anglo-Saxon countries [21,22]. Moreover, consumers eat more fish, legumes, and olive oil in Southern European countries [23]. Yet, to our knowledge, neither a tool to measure the consumer food environment nor an adaptation of the NEMS-S tool exists for these settings.

To address this gap, we have developed the Nutrition Environment Measures Survey in Stores for the Spanish setting. This study aimed to adapt and evaluate inter-rater reliability, intra-rater reliability, and construct validity of the Nutrition Environment Measures Survey in Stores adapted to the Mediterranean urban context (NEMS-S-MED).

## 2. Materials and Methods

### 2.1. Study Design and Sample

This study was part of the European-funded project “Heart Healthy Hoods”, which assesses the physical and social urban environment in relation to residents’ cardiovascular risk in Madrid [24]. As part of this larger project, we adapted the original NEMS-S survey for the Mediterranean context using the city of Madrid as an example. We did not require any institutional review board for this study because no human participants were involved.

We used census tracts to define our audit areas. For reference, census tracts (called census sections in Spain) are the smallest administrative unit for the dissemination of statistical and cartographic information in Spain, in which demographic and socioeconomic data are released. Our study sample included 21 census tracts scattered around the city and encompassed a socioeconomically diverse sample. While details on this sampling strategy have been published elsewhere [25], Table S1 shows the descriptive statistics for these audit areas.

### 2.2. Measurement

#### 2.2.1. Development of the NEMS-S-MED

We used the Nutrition Environment Measures Survey in Stores for Mediterranean contexts (NEMS-S-MED) to assess the availability and affordability of healthy foods in each food store. We adapted the NEMS-S-MED from the original NEMS-S, developed by Glanz et al. [13].

The original NEMS-S was developed to assess the availability and cost of healthier options versus less-healthy options over 10 food groups based on a mainstream North American diet [12,13]. To assess these two dimensions of food access in Madrid, we made small adjustments following the recommendations found on the NEMS protocols. These adjustments were also pilot-tested before to make sure that our adapted tool did not disturb the categories or scoring structure of the original NEMS-S instrument.

During modification, we considered the applicability of the NEMS-S instrument to the Mediterranean context by referring to the latest update of the Mediterranean Diet Pyramid, the most recent dietary guidelines for the Spanish setting, and current purchasing patterns of the Spanish population [23,26,27]. As such, we included additional food items not found in the original NEMS-S: nuts, oil, legumes, or fish. We also excluded some food items from the original tool: hot dogs, frozen dinners (lasagna, roasted turkey, and meatloaf), and baked potato chips. Thus, our adapted NEMS-S-MED instrument finally included 12 food groups: (1) fresh fruits; (2) vegetables; (3) nuts; (4) non-alcoholic beverages; (5) bread, cereals, and baked goods; (6) milk and dairy products; (7) eggs; (8) oil and butter; (9) rice; (10) legumes; (11) meat and meat products; and (12) fish and fish products (shown in Table 1). The complete instrument is available in the Table S2.

We operationalized the availability of food items using a variety of items in each food category. For example, we counted separately all available varieties of the same kind of fruit. We evaluated affordability through the price (either per grams or per item if sold only by the piece) and without considering promotion prices. Data collectors first recorded if a healthier option was available for each food category (e.g., 100% juice) and then, compared its price with the regular option (e.g., juice drinks).

The outcome of the original NEMS-S score ranges from −8 to 50 [12]. NEMS-S availability scores are based on the number of different varieties of fruits and vegetables, as well as the presence of healthier options within each food category (e.g., low-fat/skimmed milk). NEMS-S price scores are based on the relative price of the healthier option within each food category (e.g., skimmed milk vs. whole milk). In addition, it assigns negative values if the cost of healthier options is greater than the cost of the comparable regular option. We modified this scoring to fit the NEMS-S-MED measures based on the original protocol. Table 2 depicts the NEMS-S-MED scoring, which ranges from 0 to 49.

#### 2.2.2. Audits

The training for the raters included taking the NEMS training modules [12], discussing with the research team on each food category and item included in the NEMS-S-MED, and studying a detailed protocol on the NEMS-S-MED. Raters also piloted the audit tool for a week.

Two raters completed all surveys on weekdays, between November and December 2019. They walked a predefined route within each census tract to guide the fieldwork. They assessed all food stores present in the census tracts and classified them into the following categories, adapted from Glanz et al. to the Mediterranean context [21,24] as supermarkets (including discounters), convenience stores (including gas stations), or traditional/specialized stores (e.g., fruit and vegetable stores, butcheries, bakeries, or fishmongers). Further, raters also collected data on the business name, street address, and hours of operation. As they entered the stores, they evaluated and scored each measure based on its availability and price. First, they looked for healthier options for each food category (as listed in Table 2) and then, they compared the price with regular options. Food stores were visited and surveyed independently on the same day by two data collectors. To assess intra-rater reliability, one of the original raters who had rated the store previously reassessed all food stores within approximately 30 days after the initial observations.

We integrated our modified NEMS-S-MED audit tool into an easy and freely accessible web-based app called Open Data Kit to facilitate data collection. Thereby, data were directly uploaded from portable devices (Android smartphones) using a wireless network connection.

### 2.3. Statistical Analyses

We analyzed the data in 2020. First, we assessed inter-rater reliability calculating both kappa statistics (κ) and intra-class correlation coefficients (ICC) and their 95% confidence interval (CI). We used kappa and percent agreement for all binary and categorical store parameters (e.g., availability of food items within a food category) items and ICC for continuous variables (e.g., price of food items). According to guidelines from Landis and Koch, we used the following cutoff ranges for kappa values: 0.0–0.20 (slightly poor), 0.21–0.40 (fair), 0.41–0.60 (moderate), 0.61–0.80 (substantial), and 0.81–1.00 (almost perfect) [28]. An ICC less than 0.4 indicated poor inter-rater reliability and ICC greater than 0.75 indicated good inter-rater reliability [29].

Second, we assessed construct validity. We compared the NEMS-MED scoring across types of food stores with known differences regarding the foods they carry according to the original NEMS-S and previous research [18,19]. We hypothesized that supermarkets would have greater availability of healthy foods, while convenience stores would have the lowest availability of healthy foods, based on previous research [24]. Finally, we compared the scorings across area-level socioeconomic status, by tertiles. We used Chi-square for dichotomous variables and t-test, or Kruskal–Wallis, for continuous variables (depending on the variable distribution). All data management and analyses were conducted using Stata statistical software, version 15 (StataCorp LLC, College Station, TX, USA).

## 3. Results

We identified a total of 43 food stores in the selected 21 census tracts located across the city of Madrid. We found no food stores in two census tracts. Out of the other census tracts, the number of food stores varied from one to six. Among these, 10 were supermarkets, 22 convenience stores, and 11 specialized stores (five fruit and vegetable stores, one butcher, one fishmonger, and four bakeries). From these stores, we used all 43 to assess inter-rater reliability and 42 for calculating intra-rater reliability (as one store was found to be closed at that time during the intra-rater evaluation).

### 3.1. Reliability

Inter-rater and intra-rater reliability for the availability dimension were consistently very high. As shown in Table 3, audits reported almost perfect agreement (κ > 0.80) for 30/32 items for inter-rater reliability and 22/32 items for intra-rater reliability. We found the highest inter-rater agreement (κ = 1.00) for Coke, baked goods, milk, rice, legumes, meat, and fresh fish. Raw nuts showed the lowest agreement (κ = 0.67). Likewise, intra-rater reliability was highest for baked goods, milk, white rice, legumes, poultry, meat, and fish items. Both semi-aged cheese and fresh fruits showed the lowest agreement (κ = 0.65).

Inter-rater and intra-rater reliability for the affordability dimension were also very high. As shown in Table 4, audits reported almost perfect agreement (ICC = 0.81–1.00) for all items for inter-rater reliability and 16/19 items for intra-rater reliability. We found the highest inter-rater agreement (ICC = 1.00) for fruits, vegetables, milk, poultry, and fish. Low-sugar cereals showed the lowest agreement (ICC = 0.83). Intra-rater reliability was highest for fruits, vegetables, and fish. Whole bread showed the lowest agreement (ICC = 0.71).

### 3.2. Construct Validity

Table 5 and Table 6 show the results of the construct validity. Our NEMS-S-MED tool was able to discriminate between store types and census tracts of different socioeconomic status. Data presented in Table 5 show the scores for the NEMS-S-MED across three different types of food stores. As Table 5 shows, the adapted NEMS-S-MED identifies differences in three types of food retailers that we hypothesized to be different regarding the characteristics assessed with the instrument. Supermarkets and fruit and vegetable stores, when compared to convenience stores, showed a higher availability of fruits and vegetables. In terms of healthy food affordability, supermarkets scored better than convenience stores. The total NEMS-S-MED score was also higher in supermarkets than in convenience stores or fruit and vegetable stores.

Data presented in Table 6 show the scores for the NEMS-S-MED across area-level socioeconomic status (considering the census tract where the food store was located). We found that stores located in middle- and high-SES areas were more likely to have greater availability of healthy foods than stores located in low-SES areas. In terms of the affordability score, we found a higher score in food stores located within the middle- and high-SES areas (as compared to stores located in low-SES areas).

## 4. Discussion

The present study adapted and evaluated an audit tool to measure the consumer food environment in the Spanish setting. Our results show that the adapted NEMS-S-MED instrument is a useful and reliable tool to measure the availability and affordability of healthy products in a large city like Madrid, with a Mediterranean retail food environment.

Our main findings show that the NEMS-S-MED instrument is coherent and measures a uniform construct. The overall mean NEMS-S-MED score was 20.7 (SD = 9.8) and ranged from 7 to 43. Most food items displayed a substantial or almost perfect inter-rater and intra-rater agreement; the percentage agreement across NEMS-S-MED availability items was almost perfect and kappa statistics were also very high (median κ = 1.00 for inter-rater; κ = 0.92 for intra-rater). For reference, the inter-rater kappa coefficient for the original NEMS-S ranged from 0.83 to 1.00 [13]. Likewise, the inter-rater and intra-rater agreements for the price assessment were also very high, with ICCs greater than 0.91 (mean ICC = 0.96 for inter-rater; ICC = 0.95 for intra-rater). These results are also similar to the results of the evaluation of the original NEMS-S.

Furthermore, our adapted NEMS-S-MED tool was able to discriminate between store types and census tracts of different socioeconomic status. Consistent with previous research, supermarkets appear to have greater overall availability of healthy products [8]. In Brazil, Martins et al. also noted that supermarkets obtained higher scores in their Brazilian version of the NEMS-S [19]. Unlike previous past studies, small specialized stores (like fruit and vegetable stores) showed similar availability and prices of fruits and vegetables than supermarkets. This could be explained by the high proportion of traditional food stores in Madrid [30].

The mean NEMS-S-MED score and all subcomponent mean scores (except fruits and vegetables) were significantly lower in the low-SES areas. Although a larger sample is needed to confirm these findings, Black et al. also noted this reduced availability of healthy food items in food retailers from low-SES areas in their systematic review [9].

The main differences between the original NEMS-S and our adapted version relate to the rationale for scoring and the food items included. Whereas the original NEMS-S considered the dietary guidelines for North Americans [13], our adapted version focuses more on the availability of traditional unprocessed foods and whole-grain foods, staple foods in Mediterranean diets. Thus, the adapted NEMS-S-MED does not include some original items (e.g., hot dog or baked potato chips) but increases the number of fresh food items (e.g., including fish and fish products) to capture differences in availability and variety of traditional products of the Mediterranean Diet. As such, we used the Mediterranean Diet Pyramid as a reference for the scoring system, which increases with the availability of food products such as olive oil, legumes, or fish [27].

We acknowledge that this study presents several limitations. First, the original NEMS-S tool was developed to measure the availability and cost of healthy food options within food stores; however, it does not assess other determinants of the consumer food environment like promotion and marketing strategies. Moreover, our adapted NEMS-S-MED tool should be replicated in other Mediterranean cities to ensure validity and reliability in other settings. Yet, the sample of stores included in this study (*n* = 43) is similar to previous studies adapting the NEMS-S audit tool in China, Brazil, or Canada (*n* = 20, 44, and 55, respectively) [17,18,19].

Despite these limitations, this study also has important strengths. To the best of our knowledge, this is the first study to both adapt and test the validity of the NEMS tools to evaluate the consumer food environment in Europe. Southern European food environments are of particular interest because their urban form patterns differ greatly from the more sprawled North American or Australasian cities [21,31]. Furthermore, the high inter-rater reliability scores reflect the relevance of rigorous training and consensus building before the measurement phase of the study.

Future research should aim to track changes in the retail food environment over time using combined measures (including direct observations and GIS-based measures) to facilitate the understanding of how changes in the consumer food environment may deliver changes benefitting residents of low-SES areas.

## 5. Conclusions

The adapted NEMS-S-MED instrument is a reliable audit tool to assess the consumer food environment in Mediterranean urban contexts. Like the original NEMS-S tool, our study showed almost perfect inter-rater and intra-rater agreement for availability and affordability dimensions. Furthermore, the NEMS-S-MED tool was able to discriminate between store types and census tracts of different socioeconomic status. Future research should apply this instrument in other Mediterranean cities. Adapting standardized measurement tools to assess context-dependent features of food environments may facilitate the development of effective policy interventions to increase healthy food access and affordability.

## Figures and Tables

**Table 1:** Food categories and food items included in the adapted Nutrition Environment Measures Survey in stores for Mediterranean contexts (NEMS-S-MED) audit tool.

	Variables		
Food Category	Availability	Price ^1^	
		Absolute	Comparative
1. Fresh fruits			
- Apples	X	X	
2. Vegetables (fresh/frozen)			
- Tomatoes	X	X	
- Frozen spinach	X	X	
- Potatoes	X		
3. Nuts			
- Raw vs. processed	X		
4. Non-alcoholic beverages			
- Soda: Diet Coke vs. regular Coke	X		X
- Fruit juice: 100% juice vs. juice drinks	X		X
5. Bread, cereals, and baked goods			
- 100% whole grain bread	X		
- Baked goods	X		
- Plain cereals vs. >5 g of sugar per 100 g	X		X
6. Milk and dairy products			
- Skim/low-fat vs. whole milk	X		X
- White cheese vs. aged cheese	X		
- Low-fat yoghurts	X		
7. Eggs	X		
8. Oil and butter			
- Extra virgin olive oil	X	X	
- Sunflower oil	X	X	
- Butter vs. non-added salt butter	X		
9. Rice			
- Whole rice vs. white rice	X		X
10. Legumes	X		
11. Meat and meat products (red and poultry)			
- Red meat (beef) vs. poultry (chicken)	X		X
- Processed meat	X		
12. Fish			
- Fresh fish varieties	X	X	
- Unprocessed vs. processed seafood	X		
- Canned Tuna	X		

^1^ Following Glanz et al. [12], absolute price applies when the item is compared across store type and neighborhood characteristics, while comparative price applies when there is price information for a healthier option and the “regular” comparison (e.g., diet vs. regular soda).

**Table 2:** Nutrition Environment Measurement Survey in Stores for Mediterranean contexts NEMS-S-MED scoring.

Food Group	Availability	Price
Fresh fruits	If ≤5 varieties = 2 pt If 6–10 varieties = 3 pt If >10 varieties = 4 pt	No score, to compare between store types
Vegetables	Fresh vegetables If ≤ 5 varieties = 2 pt If 6–10 varieties = 3 pt If >10 varieties = 4 pt Frozen vegetables If ≤ 5 varieties = 2 pt If 6–10 varieties = 3 pt If >10 varieties = 4 pt	No score, to compare between store types
Nuts	If raw nuts = 1 pt	No score, to compare between store types
Non-alcoholic beverages	If 100% juice = 1 pt	If diet soda ≤ regular = 2 pt If 100% juice ≤ juice drinks = 2 pt
Bread, cereals, and baked goods	If 100% whole grain bread = 1 pt If low-sugar cereals = 1 pt	If low-sugar cereals ≤ regular = 2pt
Milk and dairy products	If any milk = 1 pt If skimmed yoghurt = 1 pt If cheese available = 1 pt	If skimmed- or low-fat milk < whole milk = 2 pt If skimmed- or low-fat milk = whole milk = 1 pt
Eggs	If eggs = 1 pt	No score, to compare between store types
Oil and butter	If extra virgin olive oil = 1 pt	No score, to compare between store types
Rice	If whole rice = 2 pt If white rice = 1 pt	If whole rice ≤ white rice = 2 pt
Legumes	If legumes = 1 pt	No score, to compare between store types
Meat and meat products	If red meat = 1 pt If poultry = 2 pt	If poultry meat ≤ red meat = 2 pt
Fish	Fresh fish If ≤ 5 varieties = 2 pt If 6–10 varieties = 3 pt If >10 varieties = 4 pt Frozen fish If ≤ 5 varieties = 2 pt If 6–10 varieties = 3 pt If >10 varieties = 4 pt Canned Tuna = 1pt	No score, to compare between store types
**Score (0–49)**	**Availability: 0–37**	**Price: 0–12**

**Table 3:** Reliability for the availability dimension of the Nutrition Environment Measures Survey in stores for Mediterranean contexts (NEMS-S-MED).

	Inter-Rater Reliability (*n* = 43)		Intra-Rater Reliability (*n* = 42)	
Food Item	Percent Agreement [95% CI]	Cohen Kappa ^1^ [95% CI]	Percent Agreement [95% CI]	Cohen Kappa ^1^ [95% CI]
Fresh fruits	0.95 [0.88–1.00]	0.87 [0.70–1.00]	0.85 [0.74–0.97]	0.65 [0.40–0.91]
Fresh vegetables	0.93 [0.85–1.00]	0.82 [0.62–1.00]	0.85 [0.73–0.97]	0.67 [0.42–0.91]
Frozen vegetables	0.93 [0.85–1.00]	0.85 [0.69–1.00]	0.90 [0.80–1.00]	0.79 [0.58–0.99]
Raw nuts	0.83 [0.72–0.95]	0.67 [0.45–0.89]	0.90 [0.80–1.00]	0.76 [0.53–0.98]
Processed nuts	0.93 [0.85–1.00]	0.83 [0.65–1.00]	0.89 [0.81–0.97]	0.75 [0.55–0.94]
Diet Coke	1.00 [1.00–1.00]	1.00 [1.00–1.00]	0.97 [0.90–1.00]	0.93 [0.79–1.00]
Coke	1.00 [1.00–1.00]	1.00 [1.00–1.00]	1.00 [1.00–1.00]	1.00 [1.00–1.00]
100% juice	0.97 [0.92–1.00]	0.91 [0.75–1.00]	0.97 [0.90–1.00]	0.91 [0.74–1.00]
Juice drinks	0.97 [0.93–1.00]	0.93 [0.80–1.00]	1.00 [1.00–1.00]	1.00 [1.00–1.00]
Whole grain bread	0.95 [0.88–1.00]	0.91 [0.79–1.00]	0.85 [0.74–0.97]	0.71 [0.50–0.93]
Baked goods	1.00 [1.00–1.00]	1.00 [1.00–1.00]	1.00 [0.95–1.00]	1.00 [0.95–1.00]
Low-sugar cereals	0.95 [0.88–1.00]	0.88 [0.72–1.00]	0.93 [0.83–0.99]	0.82 [0.62–1.00]
Plain cereals	1.00 [1.00–1.00]	1.00 [1.00–1.00]	0.83 [0.70–0.95]	0.66 [0.42–0.90]
Skimmed milk	1.00 [1.00–1.00]	1.00 [1.00–1.00]	1.00 [0.95–1.00]	1.00 [0.95–1.00]
Low-fat milk	1.00 [1.00–1.00]	1.00 [1.00–1.00]	1.00 [0.95–1.00]	1.00 [0.95–1.00]
Whole milk	1.00 [1.00–1.00]	1.00 [1.00–1.00]	1.00 [0.95–1.00]	1.00 [0.95–1.00]
Low-fat yoghurt	1.00 [1.00–1.00]	1.00 [1.00–1.00]	0.85 [0.73–0.97]	0.71 [0.48–0.93]
White cheese	0.86 [0.75–0.97]	0.72 [0.52–0.92]	0.90 [0.80–1.00]	0.80 [0.62–0.99]
Semi-aged cheese	0.97 [0.92–1.00]	0.95 [0.85–1.00]	0.83 [0.70–0.95]	0.65 [0.41–0.89]
Eggs	0.97 [0.92–1.00]	0.92 [0.77–1.00]	0.97 [0.90–1.00]	0.92 [0.76–1.00]
Whole rice	1.00 [1.00–1.00]	1.00 [1.00–1.00]	0.97 [0.90–1.00]	0.91 [0.74–1.00]
White rice	1.00 [1.00–1.00]	1.00 [1.00–1.00]	1.00 [0.95–1.00]	1.00 [0.95–1.00]
Legumes	1.00 [1.00–1.00]	1.00 [1.00–1.00]	1.00 [0.95–1.00]	1.00 [0.95–1.00]
Olive oil	0.95 [0.88–1.00]	0.93 [0.83–1.00]	0.97 [0.91–1.00]	0.94 [0.84–1.00]
Sunflower oil	1.00 [1.00–1.00]	1.00 [1.00–1.00]	0.92 [0.83–0.99]	0.83 [0.64–1.00]
Butter	1.00 [1.00–1.00]	1.00 [1.00–1.00]	0.90 [0.80–1.00]	0.81 [0.62–0.99]
Red meat	1.00 [1.00–1.00]	1.00 [1.00–1.00]	1.00 [0.95–1.00]	1.00 [0.95–1.00]
Poultry meat	1.00 [1.00–1.00]	1.00 [1.00–1.00]	1.00 [0.95–1.00]	1.00 [0.95–1.00]
Processed meat	1.00 [1.00–1.00]	1.00 [1.00–1.00]	0.97 [0.90–1.00]	0.94 [0.81–1.00]
Fish	1.00 [1.00–1.00]	1.00 [1.00–1.00]	1.00 [0.95–1.00]	1.00 [0.95–1.00]
Frozen fish	0.95 [0.88–1.00]	0.87 [0.70–1.00]	0.90 [0.80–1.00]	0.77 [0.55–0.99]
Canned tuna	1.00 [1.00–1.00]	1.00 [1.00–1.00]	1.00 [0.95–1.00]	1.00 [0.95–1.00]

^1^ Kappa was calculated based on the availability of the food item (yes/no).

**Table 4:** Reliability for affordability dimension of the Nutrition Environment Measures Survey in stores for Mediterranean contexts (NEMS-S-MED).

Item	Inter-Rater Reliability (*n* = 43)		Intra-Rater Reliability (*n* = 42)	
	ICC ^1^	95% CI	ICC ^1^	95% CI
Apple	0.95	[0.92–0.98]	0.95	[0.92–0.98]
Tomato	1.00	[1.00–1.00]	1.00	[1.00–1.00]
Frozen spinach	1.00	[1.00–1.00]	0.86	[0.76–0.92]
Diet Coke	0.95	[0.92–0.98]	0.86	[0.75–0.89]
Coke	0.95	[0.92–0.98]	0.91	[0.83–0.95]
100% juice	0.91	[0.84–0.95]	0.91	[0.84–0.95]
Juice drinks	0.91	[0.83–0.95]	0.90	[0.82–0.94]
Whole bread	0.95	[0.91–0.97]	0.71	[0.53–0.84]
Low-sugar cereals	0.83	[0.70–0.90]	0.78	[0.61–0.87]
Plain cereals	0.95	[0.92–0.97]	0.77	[0.61–0.87]
Skimmed milk	1.00	[1.00–1.00]	0.95	[0.91–0.97]
Whole milk	1.00	[1.00–1.00]	0.95	[0.91–0.97]
Whole rice	0.92	[0.86–0.95]	0.92	[0.86–0.96]
White rice	0.85	[0.74–0.92]	0.85	[0.73–0.91]
Olive oil	1.00	[1.00–1.00]	0.86	[0.75–0.92]
Sunflower oil	0.95	[0.92–0.97]	0.81	[0.67–0.89]
Red meat	0.99	[0.99–1.00]	0.84	[0.72–0.91]
Poultry meat	1.00	[0.99–1.00]	0.92	[0.86–0.96]
Fish	1.00	[0.99–1.00]	1.00	[0.99–1.00]

^1^ ICC—intraclass correlation coefficient.

**Table 5:** Nutrition Environment Measurement Survey in Stores for Mediterranean contexts (NEMS-S-MED) scores by store type.

Item	Store Type			
	Supermarkets ^1^ (*n* = 10)	Convenience Stores ^1^ (*n* = 22)	Fruit and Vegetables ^1^ (*n* = 5)	*p*-Value ^2^
**Fruit and Vegetables**	11 [2]	5 [3]	9 [0]	0.0001
**Availability**	29 [11]	16 [4]	12 [1]	0.0001
**Price**	5 [2]	3 [0]	1 [0]	0.0007
**Total score**	35.5 [15]	19 [5]	13 [1]	0.0001

^1^ Median [interquartile range], ^2^ *p*-values correspond to Kruskal–Wallis test.

**Table 6:** Nutrition Environment Measures Survey in stores for Mediterranean contexts (NEMS-S-MED) scores by census tract-level socioeconomic status.

Item	Area-Level SES			
	Low-SES ^1^	Middle-SES ^1^	High-SES ^1^	*p*-Value ^2^
**Fruit and Vegetables**	5 [6]	6 [5]	6.5 [5]	0.3849
**Availability**	12 [3]	19 [12]	18 [10]	0.0128
**Price**	1 [2]	3 [2]	3 [2]	0.0471
**Total score**	14 [5]	22 [14]	19.5 [10]	0.0095

^1^ Median [interquartile range], ^2^ *p*-values correspond to Kruskal–Wallis test. SES—socioeconomic status.

## Supplementary Materials

The following are available online at https://www.mdpi.com/1660-4601/17/19/7031/s1, Table S1: Descriptive statistics of the 21 census tracts included as audit areas (Madrid, Spain), Table S2: Nutrition Environment Measures Survey in stores for Mediterranean contexts (NEMS-S-MED).
