# Supplementary material for: Adaptation and Evaluation of the Nutrition Environment Measures Survey in Stores to Assess Mediterranean Food Environments (NEMS-S-MED)
Source: Int J Environ Res Public Health. 2020 Sep 25;17(19):7031. doi: 10.3390/ijerph17197031 (PMC7579010; doi:10.3390/ijerph17197031)
Supplement: Supplementary file 1 [file ijerph-17-07031-s001.pdf]

SUPPLEMENTARY MATERIAL

Table S1. Descriptive statistics of the 21 census tracts included as audit areas (Madrid, Spain)

Census tract	Low education (%)[1]	High education (%)[2]	Manual work (%)[3]	Temporary work (%)[4]	Part-time work (%)[5]	Unemployment (%)[6]	Average housing prices (€/m²)
1	7.5	57.4	15.1	19.8	18.7	6.6	4232.4
2	14.2	35.2	20.6	22.0	22.1	8.1	2382.0
3	10.9	47.1	11.7	21.1	18.8	6.2	3763.0
4	9.4	50.3	12.7	19.8	18.6	6.4	3263.5
5	9.1	48.4	13.5	19.2	19.9	6.6	3268.2
6	7.0	55.3	13.7	18.2	17.4	6.4	3884.8
7	4.7	62.7	7.0	17.9	15.2	4.8	3717.1
18	7.8	55.2	8.1	20.6	16.2	5.5	2541.3
19	18.3	29.3	21.2	22.3	23.8	7.7	2396.5
10	26.3	18.1	29.7	23.2	26.3	9.1	1979.5
11	25.9	18.8	27.9	21.6	23.0	8.7	1812.6
12	33.2	8.5	36.6	24.6	26.0	11.0	1830.0
13	26.6	15.1	35.2	25.8	28.7	10.1	1362.0
14	15.4	44.2	21.6	16.5	14.0	8.6	2734.9
15	6.8	63.8	8.2	16.7	14.1	5.7	3800.7
16	3.7	62.2	4.7	11.7	10.9	4.1	2938.6
17	14.9	18.5	31.9	22.8	23.2	9.3	1784.5
18	4.4	38.1	20.6	18.9	16.7	8.1	2517.7
19	7.7	42.4	18.8	15.4	15.1	5.6	2520.9
20	9.1	44.6	11.3	18.5	18.6	6.5	2223.9
21	8.4	48.7	9.3	16.7	17.1	5.7	2428.4
**Mean (SD)**	12.9 (0.08)	41.1 (0.16)	18.1 (0.09)	19.7 (0.03)	19.3 (0.04)	7.2 (0.01)	2732.49 (789.21)
**Min**	3.7	8.5	4.7	11.7	10.9	4.1	1362.00
**Max**	33.2	63.8	36.6	25.8	28.7	11.0	4232.38

[1] Low education = % of people above years with primary studies or below, [2] High education = % of people above years with primary studies or below, [3] Manual work = % of people aged 16 years or over working in manual or unqualified jobs with respect to the total employed population aged 16 or over, [4] Temporary work = % people aged 16 years or over in temporary jobs, [5] Part-time work = % workers in part-time jobs, [6] Unemployment = % of residents aged 16 years or over registered as unemployed among residents aged 16–64 years, SD = Standard Deviation.

Nutrition Environment Measures Survey in Stores for Mediterranean contexts (NEMS-S-MED)

A1. Retailer ID: A unique identifier will be assigned to each food store.

A2. Retailer name: Write down the full name, as it appears on the label.

A3. Type of food retailer

1. Hypermarket

2. Supermarket

3. Discount supermarket, discounter

4. Self-Service

5. Specialized store

5.1. Fruit & vegetables store

5.2. Butcher

5.3. Fishmonger

5.4. Bakery

5.5. Herbalist, eco-store

5.6. Frozen foods store

5.7. Winery with food

6. Convenience Store

7. Other type (specify)

A4. Situation

1. Open

2. Closed - If, after two different visits, the store is still found to be closed. Please try to confirm its situation with a neighbor or trader and record it as such.

A5. Schedule: Write it down if available. Follow the instructions of the online app.

A6. Photography

A7. Fresh fruits

1. Fresh fruit

1.1. Is there fresh fruit for sale? Yes / No – If not → question A8

1.2. How many varieties of fresh fruit are on sale?

a) 0-2
b) 3-5
c) 6-8
d) 9-10
e) +10

1.3. Apple price:

a) ___, __ €/kg
b) ___, ___ €/piece
c)___, __ €/tray ; _____g by tray

A8. VEGETABLES

2. Fresh vegetables

2.1. Are there fresh vegetables for sale? Yes / No

2.2. How many varieties of fresh vegetables are on sale?

a) 0-2
b) 3-5
c) 6-8
d) 9-10
e) +10

2.3. Tomato (price):

a) ___, __ €/kg
b) ___, ___ €/piece
c)___, __ €/tray ; _____g by tray

2.4. Are there potatoes for sale? Yes / No

3. Frozen vegetables

3.1. Are there frozen vegetables for sale? Yes / No

3.2. How many varieties of frozen vegetables are on sale?

a) 0-2
b) 3-5
c) 6-8
d) 9-10
e) +10

3.3. Spinach_price:

a) ___, __ €/kg
b) ___, ___ €/piece
c)___, __ €/tray ; _____g by tray

A9. Nuts

4. Are there unprocessed nuts for sale? Yes / No

5. Are there processed nuts for sale? Yes / No

A10. Non-alcoholic beverages

6. Soda

6.1. Is there light coke for sale? Yes / No

6.2. Light coke price

a)__ , _ _ € /can (33cl)
b)__, __ €/bottle (1l)

6.3. Is there regular coke for sale? Yes / No .

6.4. Normal coke_ price

a)__ , _ _ € /can (33cl)
b)__, __ €/bottle (1l)

7. Juices

7.1. Is there 100% juice for sale? Yes / No – If not → question 13

7.2. Juice price 100%

a) ___, __ €/l
b) ___, ___ €/pack ; ____ L by pack

7.3. Is there nectar (non-100% juice) for sale? Yes No - If not → question 13

7.4. Nectar price

a) ___, __ €/l
b) ___, ___ €/pack ; ____ L by pack

A11. Bread, cereals and baked goods

8. Bread

8.1. Is there whole-grain bread for sale? Yes / No

9. Cereals

9.1. Are there low-sugar cereals (<7g sugar per serving) Yes No - If not → question 10

9.2. Low-sugar cereal price

a) ___, __ €/kg
b) ___, ___ €/pack ; ____ g pack

9.3. Are there cereals for sale? Yes / No

9.4. Cereals_price

a) ___, __ €/kg
b) ___, ___ €/pack ; ____ g pack

10. Baked goods

10.1. Are there baked goods for sale? Yes/ No

A12. Milk and dairy products

11. Milk

11.1. Is there skimmed-milk for sale? Yes / No

11.2. Price of skimmed-milk _ , _€

11.3. Is there half-skim milk for sale? Yes / No

11.4. Is there whole milk for sale? Yes / No – If not, go to question 12

11.5. Price of whole milk _ , _ _€

12. Yogurts

12.1. Are there skimmed yogurts for sale? Yes / No

13. Cheese

13.1. Is there semi-cured cheese for sale? Yes / No

13.2. Is there fresh cheese for sale? Yes / No

A13. Eggs

14.1. Are eggs for sale? Yes / No

A14. Oil and butter

15. Oil

15.1. Is there extra virgin olive oil for sale? Yes / No –If not → question 15.3

15.2. Price of extra virgin olive oil

a) __, __ €/l
b) __, __ €/pack .__, __L by pack

15.3. Is there sunflower oil for sale? Yes / No –If not → question 16

15.4. Price of sunflower oil

a) ___, __ €/l
b) __, __ €/pack .__, __L by pack

16. Butter

16.1. Is there light butter for sale? Yes / No

16.2. Is there normal butter for sale? Yes / No

A15. Rice

17. Rice

17.1. Is there brown rice for sale? Yes /No → 17.3

17.2. Price of brown rice

a) __, __ €/kg
b) __, __ €/pack ,__, __g by pack

17.3. Is there white rice for sale? Yes / No - If not, → A16

17.4. Price of white rice

a) __, __ €/kg
b) __, __ €/pack ,__, __g by pack

A16. Legumes

18. Are there legumes for sale? Yes / No

A17. Meat and meat products

19. Beef

19.1. Is there beef for sale? Yes / No –If not → A20

19.2. Price of beef steaks

a) __, __ €/kg
b) __, __ €/pack ,__, __g by pack

20. Poultry meat

20.1. Is there white meat for sale? Yes / No –If not → A21

20.2. Price chicken breast fillets

a) __, __ €/kg
b) __, __ €/pack ,__, __g by pack

21. Processed meat

21.1. Are there sausages for sale? Yes / No

A18. Fish

22. Fresh fish

22.1. Is there fresh fish for sale? Yes / No –If not → A23

22.2. How many varieties of fresh fish are on sale?

a) 0-2
b) 3-5
c) 6-8
d) 9-10
e) +10

22.3. Price of hake fillet.

a) ___, __ €/kg
b) ___, ___ €/piece
c)___, __ €/tray ; _____g by tray

23. Frozen fish

23.1. Is there unprocessed frozen fish for sale? Yes/ No → A24

23.2. How many varieties of frozen fish are on sale?

a) 0-2
b) 3-5
c) 6-8
d) 9-10
e) +10

23.3. Is there frozen fish processed for sale? Yes / No

24. Fish preserves

24.1. Is there tuna for sale? Yes/ No
